# Silicon diffusion control in atomic-layer-deposited Al_2_O_3_/La_2_O_3_/Al_2_O_3_ gate stacks using an Al_2_O_3_ barrier layer

**DOI:** 10.1186/s11671-015-0842-2

**Published:** 2015-03-19

**Authors:** Xing Wang, Hong-Xia Liu, Chen-Xi Fei, Shu-Ying Yin, Xiao-Jiao Fan

**Affiliations:** Key Laboratory for Wide-Band Gap Semiconductor Materials and Devices of Education, School of Microelectronics, Xidian University, No. 2, South Taibai Road, Yanta Zone, Xi’an City, Shaanxi Province 710071 People’s Republic of China

**Keywords:** Atomic layer deposition, Diffusion, Interfacial layer, Silicate, Equivalent oxide thickness

## Abstract

In this study, the physical and electrical characteristics of Al_2_O_3_/La_2_O_3_/Al_2_O_3_/Si stack structures affected by the thickness of an Al_2_O_3_ barrier layer between Si substrate and La_2_O_3_ layer are investigated after a rapid thermal annealing (RTA) treatment. Time of flight secondary ion mass spectrometry (TOF-SIMS) and X-ray photoelectron spectroscopy (XPS) tests indicate that an Al_2_O_3_ barrier layer (15 atomic layer deposition (ALD) cycles, approximately 1.5 nm) plays an important role in suppressing the diffusion of silicon atoms from Si substrate into the La_2_O_3_ layer during the annealing process. As a result, some properties of La_2_O_3_ dielectric degenerated by the diffusion of Si atoms are improved. Electrical measurements (*C*-*V*, *J*-*V*) show that the thickness of Al_2_O_3_ barrier layer can affect the shift of flat band voltage (*V*_FB_) and the magnitude of gate leakage current density.

## Background

Microelectronics technology has developed in accordance with Moore’s law for many years. The performance of metal-oxide-semiconductor field-effect transistor (MOSFET) has been improving with the downscaling of feature size. However, in sub-45-nm complementary metal oxide semiconductor (CMOS) technology, the scaling of SiO_2_ gate dielectric thickness leads to an unacceptable gate leakage current, which affects the reliability of the device and causes an increase in static power dissipation. Therefore, new kinds of dielectric materials with high permittivity are needed to replace the traditional SiO_2_ gate dielectric to obtain a smaller equivalent oxide thickness (EOT) in the CMOS industry [1,2]. Presently, the use of HfO_2_ (*k* ~ 13 to 20) as the gate dielectric in the high-*k*/metal gate structure has been successfully applied to MOSFET fabrication and is gradually replacing the traditional SiO_2_/poly-Si gate structure [3]. Nevertheless, further downscaling trend makes the use of HfO_2_ as gate dielectric in the CMOS technology encounter a bottleneck. During the past two decades, rare earth oxides (Y_2_O_3_, La_2_O_3_, Nd_2_O_3_, etc.) used as alternative gate dielectric materials have been extensively studied [4]. In particular, due to its high k value (approximately 27) and large band gap (approximately 5.3 eV), lanthanum oxide (La_2_O_3_) is considered as one of the most promising alternative for HfO_2_ to achieve a more aggressive downscaling of the EOT [5]. But disadvantages of La_2_O_3_ have also been reported, such as hygroscopicity and affinity for silicon atoms [6]. Al_2_O_3_ has also been used as high-*k* gate dielectric material in the early stage, but its further development is limited because of the low *k* value (8 to 10). However, the combination of Al_2_O_3_ and La_2_O_3_ results in an improvement in the characteristics of the films used as the gate dielectric. For example, when the La_2_O_3_ layer is *in situ* capped with an Al_2_O_3_ layer, the absorption of moisture which gives rise to detrimental effects on the dielectric films such as increased surface roughness and deterioration of the permittivity can be suppressed [7]; in addition, as a compound of Al_2_O_3_ and La_2_O_3_, LaAlO_3_ (LAO) has a nearly high k value (25 to 27) as La_2_O_3_ while providing a high immunity against moisture absorption and a preferable thermal stability [8] during the annealing process.

It turns out that an interfacial layer (IL) which exhibits a La-silicate composition is unavoidably formed between the La_2_O_3_ film and Si substrate. Moreover, in the conventional gate first process with a high-temperature annealing treatment, the diffusion of Si atoms from the substrate into the dielectric results in the formation of undesirable low-permittivity silicates in the films. Both of the two phenomena mentioned above can especially be observed in low-temperature deposited films in which the existence of large amounts of defects and disordered chemical bonds may enhance the diffusion of oxygen and Si atoms and lead to a degradation of the EOT value [9]. Atomic layer deposition (ALD) is a typically low-temperature deposition method, but its self-limited surface reaction mechanism makes the films deposited by ALD have some outstanding properties such as atomic scale thickness controllability, fine uniformity, and excellent conformality [10]. Regarding this, ALD is considered as one of the most appropriate way to produce high-quality high-*k* gate dielectric. In this study, we prepared Al_2_O_3_/La_2_O_3_/Al_2_O_3_ gate stacks by ALD to circumvent the hygroscopicity and diffusion-related problems of La_2_O_3_. A thickness-varied Al_2_O_3_ layer was deposited between La_2_O_3_ layer and Si substrate as a barrier layer, and its effects on the physical and electrical characteristics of the films were investigated.

## Methods

Al_2_O_3_/La_2_O_3_/Al_2_O_3_ gate stacks (S1 ~ S4) were deposited on p-type Si (100) wafers using an atomic layer deposition reactor (Picosun R-200, Espoo, Finland). The wafers were treated with a diluted HF solution to remove the native SiO_2_ before deposition. La(^i-^PrCp)_3_ and TMA were used as the La and Al precursor while O_3_ was used as the oxidant. Under the deposition temperature of 300°C, for La_2_O_3_, a linear relation with a growth rate of approximately 0.85 Å/cycle is obtained, and the steady-state growth rate of Al_2_O_3_ films is approximately 0.93 Å/cycle. The fabricated nanolaminate (Al_2_O_3_/La_2_O_3_/Al_2_O_3_) films were annealed at 700°C for 1 min in N_2_ ambient. Film thicknesses were measured by Woollam M2000D (Woollam Co. Inc., Lincoln, NE, USA) spectroscopic ellipsometry (SE). Cross-sectional high-resolution transmission electron microscopy (HRTEM) performed with the [100] direction [11] of the Si substrate was used to observe the microstructures of the gate stacks. The bonding structures and chemical quantitative composition of the films were examined by X-ray photoelectron spectroscopy (XPS) and time of flight secondary ion mass spectrometry (TOF-SIMS). The electrical properties of the films were measured using a metal-insulator-semiconductor (MIS) capacitor structure. Metal gate (160 nm Au/20 nm Ni) with a diameter of 300 μm was deposited by magnetron sputtering through a shadow mask, and Al was sputtered as the back contact metal, followed by annealing in forming gas ambient at 400°C for 20 min. The capacitance-voltage (*C*-*V*) and leakage current density-voltage (*J*-*V*) measurements were carried out using a Keithley 590 C-V analyzer (Keithley Instruments, Cleveland, OH, USA) and HP 4156B instrument (Hewlett-Packard Development Company, L.P., Palo Alto, CA, USA). The flat band voltages (*V*_FB_) and EOT of the capacitors were extracted from the simulation software named Hauser NCSU CVC program [12] taking into account of quantum-mechanical effects.

## Results and discussion

**Figure 1:**
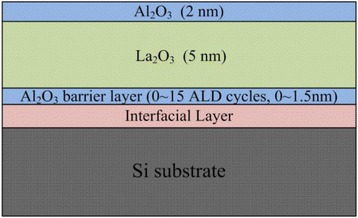
**Schematic structure of Al**_**2**_ **O**_**3**_ **/La**_**2**_ **O**_**3**_ **/Al**_**2**_ **O**_**3**_ **/Si stacks.** The thickness of Al_2_O_3_ barrier layer between Si substrate and La_2_O_3_ layer in samples S1 ~ S4 changes with the number of ALD cycles.

Figure 1 shows the schematic structure of Al_2_O_3_/La_2_O_3_/Al_2_O_3_ gate stacks discussed in this paper. The thickness of La_2_O_3_ layers in samples S1 ~ S4 is 5 nm, and all the samples are *in situ* ALD-capped with a 2-nm Al_2_O_3_ layer. The thickness of the Al_2_O_3_ barrier layer between La_2_O_3_ layers and Si substrate is tuned by varying the number of ALD cycles, which is 0, 5, 10, and 15 cycles for samples S1 ~ S4 separately. Table 1 shows the total thickness of the as-grown and annealed samples measured by spectroscopic ellipsometry (SE). It is reported that La_2_O_3_ films are easily hydrated, but in this work, the existence of capping Al_2_O_3_ layer suppresses the hydration reaction during the rapid thermal annealing (RTA) process, and as a result, the thickness of each sample does not increase too much after the annealing treatment. However, sample S1 shows a thicker increment about 0.4 nm when compared with the other three samples, which indicates a thicker interfacial layer formation in sample S1.

**Table 1:** **Thickness (measured by SE) of the as-grown and annealed samples discussed in this work**

**Sample**	**Number of ALD Al**_**2**_ **O**_**3**_ **cycles**	**Thickness as-grown (nm)**	**Thickness after RTA (nm)**
S1	0	8.2	8.6
S2	5	8.4	8.7
S3	10	8.8	9.0
S4	15	9.3	9.5

**Figure 2:**
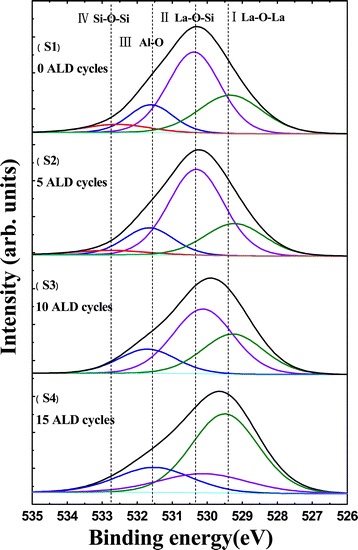
**O 1s XPS spectra of Al**_**2**_ **O**_**3**_ **/La**_**2**_ **O**_**3**_ **/Al**_**2**_ **O**_**3**_ **gate stacks shown in Table** 1 **.**

Figure 2 shows the O 1s XPS spectra and their deconvolution results for the RTA treated Al_2_O_3_/La_2_O_3_/Al_2_O_3_ gate stacks. C 1s peak from adventitious carbon at 284.6 eV [13] was used as an internal energy reference during the analysis. The O 1s XPS spectra consists of four peaks, which are (I) La-O-La (approximately 529.3 eV), (II) La-O-Si (approximately 530.4 eV), (III) Al-O (approximately 531.6 eV), and (IV) Si-O-Si (approximately 532.6 eV) [14]. Among the four deconvoluted peaks, peak I and peak III come from the deposited La_2_O_3_ and Al_2_O_3_ layer; peak II and peak IV are attributed to SiO_x_ and La-silicate, which indicate the formation of interfacial layer and silicate in the films caused by the diffusion of oxygen and Si atoms during the annealing process [15]. By contrast, we can find out that the intensity of peak II and peak IV corresponding to La-O-Si and Si-O-Si is reduced from samples S1 to S4, respectively. The decreasing trend of peak II and peak IV suggests that, on the one hand, in sample S1 without an Al_2_O_3_ barrier layer, Si diffusion into the La_2_O_3_ layer is enhanced during a thermal treatment, resulting in a favorable silicate formation through the film and the interface; on the other hand, to some extent, a uniform and dense Al_2_O_3_ barrier layer (approximately 1.5 nm) in sample S4 produced by 15 cycles of ALD deposition suppresses the formation of IL and prevents the diffusion of Si atoms, resulting in a silicate formation decrease. In order to study this phenomenon more clearly, cross-sectional HRTEM and time of flight secondary ion mass spectrometry (TOF-SIMS) measurements were applied to samples S1 and S4.

**Figure 3:**
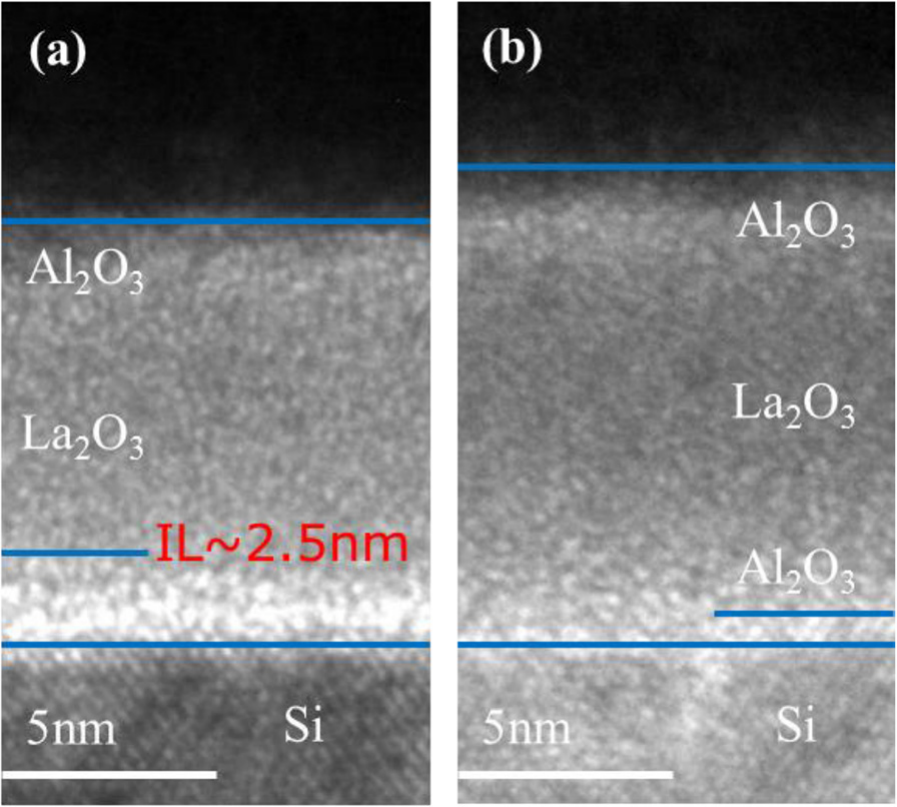
**Cross-sectional HRTEM images of annealed samples S1 and S4 shown in Table** 1 **. (a)** Sample S1. **(b)** Sample S4.

**Figure 4:**
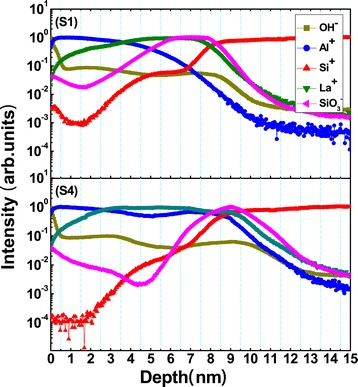
**TOF-SIMS depth profiles of annealed samples S1 and S4 shown in Table** 1 **.**

With the deposition method of ALD, different from the LaLuO_3_/Si stack, in which a sharp interface is observed [16], the IL seems hardly to be avoided in the La_2_O_3_/Si stack because of the affinity for silicon atoms of La_2_O_3_, especially after a high-temperature RTA treatment. The cross-sectional HRTEM images for annealed samples S1 and S4 are displayed in Figure 3. Both of the films exhibit an amorphous structure up to an annealing temperature of 700°C [17]. Compared with Figure 3b, a thicker amorphous transition region about 2.5 nm between the deposited film and Si substrate is observed in Figure 3a, indicating a thicker IL formation in sample S1. Figure 4 shows the TOF-SIMS depth profiles of OH^−^, Al^+^, Si^+^, La^+^, and SiO_3_^−^ clusters acquired for samples S1 and S4. The intensity of the signals is dealt with normalization method, and depth values are calibrated by HRTEM results. Large amounts of OH^−^ are detected only at the surface of the films while the internal content is much less and uniformly distributed without a gradient distribution trend suggesting that the diffusion of moisture from air to the films is suppressed by the capping Al_2_O_3_ layer. As a result, the moisture absorption of La_2_O_3_ layer can be neglected in the Al_2_O_3_/La_2_O_3_/Al_2_O_3_ gate stacks [7]. The intensity of Si^+^ in sample S4 is at least an order magnitude less than that in sample S1, which suggests that the deposited Al_2_O_3_ barrier layer (15 cycles) in sample S4 indeed suppresses the diffusion of Si atoms from the Si substrate into the La_2_O_3_ layer during the thermal process, in good agreement with the XPS results. Due to the formation of pinholes in the Al_2_O_3_ barrier layer during the 700°C RTA treatment [18], the diffusion of Si atoms is not completely suppressed. HRTEM analysis reveals the existence of a thicker IL in the sample without an Al_2_O_3_ barrier layer, and now this result can be further confirmed from the intensity of SiO_3_^−^ signals which indicate the presence of a SiO_x_-like component existing in the region of the nanolaminate/substrate interface. Finally, the depth profiles of Al^+^ and La^+^ suggest a serious interdiffusion of La_2_O_3_ and Al_2_O_3_ layers in the Al_2_O_3_/La_2_O_3_/Al_2_O_3_ gate stacks. This result explains the difficulty in distinguishing the borderlines between La_2_O_3_ and Al_2_O_3_ layers in Figure 3.

**Figure 5:**
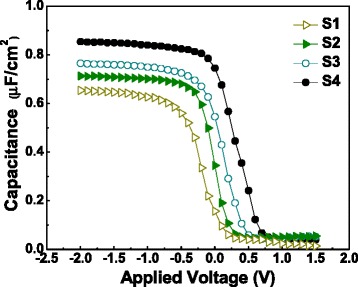
***C*** **-** ***V*** **curves of MIS capacitors using annealed S1 ~ S4 gate stacks as insulators.** The capacitors were measured at the frequency of 1 MHz.

Figure 5 shows the *C*-*V* curves and *V*_FB_ shifts for the Al_2_O_3_/La_2_O_3_/Al_2_O_3_ gate stacks. The EOTs of samples S1 ~ S4 extracted by NCSU CVC program are 4.62, 3.82, 3.85, and 3.28 nm, then the *k* values can be figured out as 7.3, 8.9, 9.1, and 11.3, respectively. For sample S1, the enhanced diffusion of oxygen and Si atoms during the annealing process owing to the absence of Al_2_O_3_ barrier layer makes the permittivity lower while the EOT thicker with respect to those of the others. The doping concentration of the Si substrate used in this work is 5.0 × 10^15^ cm^−3^, considering the work function difference between Si substrate and Au/Ni metal gate electrode, the ideal *V*_FB_ can be worked out as −0.06 V. It is believed that the shift of *V*_FB_ originates from the existence of net oxide charges in the films [19]. The *V*_FB_ value for sample S1 is −0.16 V. Accordingly, there are positive net oxide charges in sample S1. The *V*_FB_ value for samples S2 ~ S4 are observed to be 0.05, 0.15, and 0.26 V, respectively. Such *V*_FB_ shifts suggest the presence of negative net oxide charges induced by the negative fixed charges existing in the Al_2_O_3_ barrier layers [20]. Taking the ideal *V*_FB_ as a reference, a bigger shifting value of *V*_FB_ is obtained in sample S4 compared with those of sample S2 and sample S3, which means that, in a very thin range, more negative net oxide charges in the Al_2_O_3_/La_2_O_3_/Al_2_O_3_ gate stacks would be generated as the thickness of Al_2_O_3_ barrier layer increases.

**Figure 6:**
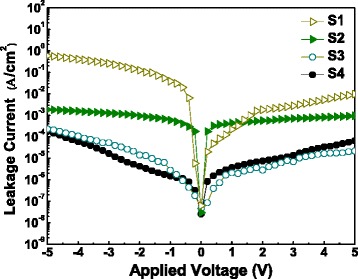
***J*** **-** ***V*** **curves of MIS capacitors using annealed S1 ~ S4 gate stacks as insulators.**

The effect of Al_2_O_3_ barrier layer thickness on the gate leakage current density is shown in Figure 6. At the applied gate voltage of −5 V, the leakage current density of the films are measured to be 5.92 × 10^−1^, 1.86 × 10^−3^, 2.32 × 10^−4^, and 1.79 × 10^−4^ A/cm^2^, separately. The current density reduction of sample S4 by three orders or more from sample S1 is achieved. The low gate leakage current characteristic of sample S4 is considered arise from the large band offsets [21] at the nanolaminate/Si interface. Al_2_O_3_ has a large band gap of 8.8 eV and high values of conduction band offset (approximately 2.8 eV) and valence band offset (approximately 4.8 eV) with respect to p-type Si substrate [22,23]. Consequently, the addition of an Al_2_O_3_ barrier layer contributes to the formation of a higher potential barrier at the fabricated nanolaminate/Si interface than that at the La_2_O_3_/Si interface or the SiO_x_-silicate/Si interface. The high potential barrier formed between the oxide film and the Si substrate results in a weakening of the tunneling effect of electrons and holes in the metal-insulator-semiconductor capacitor. Therefore, the leakage current density decreases with the existence of an Al_2_O_3_ barrier layer.

## Conclusions

In summary, an Al_2_O_3_ barrier layer (15 ALD cycles, approximately 1.5 nm) between the La_2_O_3_ layer and Si substrate plays an important role in blocking the diffusion of Si atoms from Si substrate into the La_2_O_3_ layer and the diffusion of oxygen in the opposite direction resulting in a decrease in the thickness of IL and the formation of La-silicate in the Al_2_O_3_/La_2_O_3_/Al_2_O_3_ gate stack during the annealing process. In other words, the existence of the Al_2_O_3_ barrier layer provides a gate stack with high permittivity and contributes to the achievement of an improved EOT value. The thickness of the Al_2_O_3_ barrier layer also affects the electrical characteristics of the fabricated nanolaminates. In a very thin range (0 ~ 15 cycles), Al_2_O_3_ barrier layer brings in negative net oxide charges which leads to a positive shift of *V*_FB_. In addition, as the thickness of Al_2_O_3_ barrier layer increases, gate leakage current is reduced due to the formation of a high potential barrier between the oxide film and Si substrate.

## Abbreviations

ALD: atomic layer deposition
CMOS: complementary metal oxide semiconductor
EOT: equivalent oxide thickness
HRTEM: high resolution transmission electron microscopy
IL: interfacial layer
MIS: metal-insulator-semiconductor
MOSFET: metal-oxide-semiconductor field-effect transistor
RTA: rapid thermal annealing
SE: spectroscopic ellipsometry
TOF-SIMS: time of flight secondary ion mass spectrometry
XPS: X-ray photoelectron spectroscopy
